# Healthcare Utilisation of Moluccans in the Netherlands: Equal Care for Equal Need after 60 Years of Residence in the Host Country?

**DOI:** 10.3390/ijerph17238710

**Published:** 2020-11-24

**Authors:** Adee Bodewes, Charles Agyemang, Karien Stronks, Anton E. Kunst

**Affiliations:** Department of Public and Occupational Health, Amsterdam UMC, University of Amsterdam, 1105 Amsterdam, The Netherlands; c.o.agyemang@amsterdamumc.nl (C.A.); k.stronks@amsterdamumc.nl (K.S.); a.e.kunst@amsterdamumc.nl (A.E.K.)

**Keywords:** healthcare utilisation, Moluccans, migrants, healthcare

## Abstract

Background: In many countries, recent migrants have difficulties using healthcare to the same extent as host populations. It is uncertain whether these differences persist for long-settled migrants. This study examined healthcare utilisation of Moluccans in 2012, more than 60 years after they migrated from Indonesia to the Netherlands. Methods: A survey was held among 715 Moluccans and 3417 Dutch persons. Differences in healthcare utilisation were assessed using regression analyses adjusting for age, gender, indicators of health, religious affiliation, and education. Results: Moluccans had lower rates of healthcare use, including visits to the general practitioner (odds ratio (OR) = 0.67), outpatient medical specialist (OR = 0.50), dentist (OR = 0.65), and physiotherapist (OR = 0.56), as well as the use of paid housekeeping services (OR = 0.37). Among those who visited a healthcare service, no difference was found between Moluccans and Dutch in the frequency of visits, except for physiotherapist visits (rate ratio (RR) = 0.51). For the risk of hospitalisation, no difference was found; however, of those admitted to the hospital, the frequency of admission was lower among Moluccans than Dutch (RR = 0.74). Conclusions: Despite their long residence in the host country, equal utilisation of healthcare services has not been achieved for Moluccans in the Netherlands. Demand-based factors (e.g., family networks, health beliefs, and use of traditional medicine) may contribute to the persistence of such differences and require further investigation.

## 1. Introduction

Differences in healthcare utilisation exist between migrants who originate from low/mid-income countries and their host populations, even in host countries with universal healthcare [1,2,3,4]. These differences are a serious concern, as they may indicate unequal healthcare for migrants.

In the Netherlands, healthcare utilisation among relatively recently migrated groups is generally low in comparison to their healthcare needs, at least for some healthcare services [4,5]. Although these groups tend to visit the general practitioner (GP) and use prescribed medication about as often as the Dutch population, or even more, they make less use of preventive and specialised healthcare services [4,5]. Such lower use may be due to demand-side factors (e.g., lower propensity to use preventive services) and supply-side factors (e.g., limited accessibility of specialised healthcare services). 

In the context of equity in healthcare, the principle of horizontal equity refers to equal treatment of equals. It is uncertain whether levels of healthcare utilisation among migrants become more in accordance with their health-related needs as the length of stay in their host country increases. Differences in healthcare utilisation in relation to need may persist due to cultural differences between migrants and the host population [6,7]. However, due to the relatively recent migration history of most migrant groups from low/mid-income countries, there is little evidence within Europe on the persistence or convergence of such differences many decades after immigration.

The Moluccans, who originated from the Moluccan Islands in Indonesia, provide an opportunity to gain insight into the healthcare utilisation pattern of a migrant group long after their settlement in the host country [8,9,10,11]. A unique feature of this ethnic minority group is its long migration history [8]. The Moluccans arrived in the Netherlands around 1951 with approximately 3000 soldiers and their relatives who served in the Royal Dutch East-Indies Army during the 1940s [8]. As no significant second immigration occurred since 1951, Moluccans share a common migration and integration history [10]. The total number of Moluccans is estimated today to be approximately 50,000. In general, Moluccans appear to be well-integrated in Dutch society but have a lower socioeconomic status compared to the Dutch population [8,10]. A few studies have found higher rates of overweight, hypertension, ischemic heart disease, and increased all-cause mortality among Moluccans as compared to the Dutch [9,10,12]. However, data on healthcare utilisation among this group are lacking. 

This exploratory study therefore aimed to determine differences in healthcare utilisation between Moluccans living in the Netherlands and the Dutch host population in order to assess whether their healthcare use is in accordance with their health need. We had no a priori hypothesis. On the one hand, their general use of healthcare could be in accordance to need, considering their long migration history and apparent assimilation. On the other hand, some forms of healthcare use might be lower than expected, thus resembling the patterns observed for more recent migrant groups. 

## 2. Materials and Methods 

### 2.1. The Survey

The “Moluccans & Health” study was carried out in 2012 [8]. The overall aim was to map the health status and healthcare use of Moluccans in the Netherlands. In this study, we defined Moluccan migrants as all persons who (1) lived in the Netherlands by the time of the study and (2) were born at the Moluccan Islands in Indonesia or of whom one or more parents/grandparents were born there, and (3) identified him/herself as Moluccan or mixed Moluccan-Dutch. The study aimed to obtain representative estimations, on the national level, regarding the Moluccan’s health status. For this study, the Medical Ethical Committee of the Academic Medical Centre (AMC) deemed that no approval was needed, as the Medical Research Involving Human Subjects Act (WMO) did not apply to this study (AMC: document number W12_180). All data were anonymised and stored in the institution repository of the AMC in a secured internal environment.

The POLS (Continuous Survey of Living Conditions) questionnaire of Statistics Netherlands was used as the baseline for our questionnaire. Specific questions were added regarding traditional diet and medical practices. The questionnaire was developed in Dutch and translated into the Malay language. Of the 60 Moluccan districts throughout the Netherlands, to which all Moluccans were relocated by the Dutch government during the years after their arrival, 19 were selected in order to represent variations between districts in (i) population size, (ii) religious affiliation, and (iii) geographic location within the Netherlands. Moluccan districts are mainly located at the outskirt of Dutch towns where all necessary extramural services and where hospitals can be reached within the 45-min window. Key informants of the local Moluccan organisations provided address lists of all Moluccan residents of their own Moluccan district. In each Moluccan district, all Moluccan residents are registered by these local organisations. Persons with Moluccan roots aged 30–65 years (who mainly belong to the second- and third-generation Moluccans) living in and close to one of the selected Moluccan districts were invited to participate. Questionnaires were personally handed over within each Moluccan district, door-to-door, by one of the researchers (AB) or by key informants. The respondents could fill in the questionnaire via the internet, via a personal face-to-face interview, or via the paper-and-pencil method, using either the Dutch or Malay version. Of the 2956 Moluccans who were invited to participate, 715 responded to the questionnaire (response rate: 24%) [8].

As a control group, we selected a sample of 3387 persons with Dutch nationality, matched by age, gender, and Dutch origin, who filled in the POLS (Continuous Survey of Living Conditions) health questionnaire in the year 2011. This POLS included questions on health, lifestyle, medical contacts, and preventive behaviour of the population in the Netherlands. Each year, this survey is conducted among a random sample of the Dutch population selected from municipal registries [13]. Every respondent has basic health insurance, as health insurance is obliged by law in the Netherlands.

### 2.2. Healthcare Utilisation 

The questionnaire included questions on the several aspects of healthcare utilisation: (i) contact with a GP, (ii) contact with an outpatient medical specialist, (iii) contact with a dentist, (iv) contact with a physiotherapist, (v) contact with an alternative medicine practitioner, (vi) hospital admission, (vii) use of prescribed and nonprescribed medication, and (viii) use of paid housekeeping and personal care services. Respondents were asked if they used these healthcare services in the past 2 weeks, 4 weeks, or 12 months by answering “yes” or “no”. Respondents who answered with “yes” were asked the number of visits or admissions in the past 2 weeks, 4 weeks, or 12 months, depending on the type of healthcare service. 

### 2.3. Covariates 

Based on the POLS questionnaire, we measured several sociodemographic variables, including age, gender, education level, and religious affiliation. A person’s religious beliefs were determined as (i) Christian, (ii) Islamic, (iii) other, or (iv) no religious affiliation. Education level was classified into (i) primary education, (ii) secondary education, (iii) secondary vocational education, and (iv) higher education. 

Health status was measured by questions on chronic disease prevalence, mental health, self-reported health status, and disability. The occurrence of chronic diseases was determined using a list of 16 common chronic diseases. The respondents were asked to report having suffered from one or more of the chronic diseases in the previous 12 months by answering “yes” or “no” [14]. Mental health status was determined using the Mental Health Inventory (MHI-5) scale. The MHI-5 scale comprises five questions about feeling (i) nervous, (ii) calm, (iii) energetic, (iv) miserable, and (v) so bad that nothing could cheer you up. Respondents could give six possible answers (scored from 1–6), with a total sum score ranging from 5–30. The total score was transformed to obtain a final score between 0–100 [15]. A good mental health status was represented by a final score ≥ 80, a moderate mental health status by a score of 60–80, and a poor mental health status by a score of ≤60. 

Self-reported health status was measured by the question “In general, how would you rate your health?” with answers “excellent”, “very good”, “good”, “moderate”, and “bad”. The OECD (Organisation for Economic Cooperation and Development) indicator was used to determine disabilities with hearing (2 items), eyesight (2 items), mobility (3 items), and communication (1 item). Respondents reported four possible levels of disability: “with no effort”, “with some effort”, “with a lot of effort”, or “I cannot do this” [16]. A respondent was considered disabled if one or more of the items was answered by “with a lot of effort” or “I cannot do this”. 

### 2.4. Statistical Analysis

Differences in demographic and health characteristics were examined using the chi-square and Mann-Whitney test. Binary logistic regression for binominal outcomes and Poisson regression for count data were performed to investigate differences in healthcare utilisation between Moluccans and the Dutch population. The regression models included five covariates: age, gender, indicators of health, religious affiliation, and education level. These can be considered as confounders or mediators to the association between ethnic background and healthcare utilisation. Three models were applied: Model 1 adjusted for age and gender, Model 2 additionally adjusted for indicators of health, and Model 3 additionally adjusted for religious affiliation and education level. Data were analysed using SPSS statistics for Windows (IBM Corp. SPSS Statistics for Windows, Version 19.0. Armonk, NY, USA). A *p*-value ≤ 0.05 was considered to indicate statistical significance.

### 2.5. Ethics approval and informed consent: 

Ethical approval was obtained from the Medical Ethics Review Committee of the Academic Medical Centre (AMC) Amsterdam (document number: W12_180). Participants expressed their informed consent in this study by filling in and resending the questionnaire to the researchers. In the letter accompanying the survey, participants were informed on the use of the data and publication of the results, with the guarantee of confidentiality and anonymity.

## 3. Results 

Table 1 shows the demographic characteristics for both populations. There are slightly more women (Moluccan: 51.9% and Dutch: 52.6%) in the samples than men (Moluccan: 48.1% and Dutch: 47.4%) in both populations. The majority of the Moluccan respondents were aged ≥ 50 years. Less Moluccans (16.6%) completed a higher education as compared to the Dutch (33.6%). Of all Moluccans, more than 90 percent considered themselves to be Christians, as compared to almost one-half of the Dutch.

Table 2 shows the health characteristics of the Moluccans and the Dutch population. Moluccans more often suffered from chronic diseases than the Dutch. Moluccans had higher prevalence rates for chronic diseases such as varicose veins, asthma, eczema, arthritis, and hand and wrist problems but less back problems than the Dutch population (results not shown). Similarly, compared to the Dutch, more Moluccans reported moderate-to-poor mental health, moderate-to-poor general health, and disabilities such as limited eyesight and mobility.

Table 3 presents an overview of healthcare use in both populations. After adjusting for health indicators (Model 2), Moluccans had significantly lower odds for the use of healthcare services, such as a GP (odds ratio (OR) = 0.73), outpatient medical specialist (OR = 0.52), dentist (OR = 0.72), physiotherapist (OR = 0.62), and alternative medicine practice (OR = 0.61) compared with the Dutch. Moluccans were less likely to use prescribed and nonprescribed medication (OR = 0.60 and OR = 0.56, respectively) compared to the Dutch population. Moreover, they made less use of paid housekeeping services than the Dutch (OR = 0.34). Moluccans were equally likely as the Dutch population to be admitted to a hospital (OR = 1.05). These differences remained after further adjustments for the education level and religious affiliations (Model 3). 

Table 4 shows the mean frequency of visits to healthcare services for those who visited the respective service at least once. Compared to the Dutch population, Moluccans had a slightly higher frequency of visits to a GP (mean of 1.56 vs. 1.42), outpatient medical specialist (mean of 1.78 vs. 1.55), and dentist (mean of 1.38 vs. 1.20). Moluccans had a much lower frequency of visits to a physiotherapist (mean of 12.67 vs. 26.06) and admissions to the hospital (mean of 1.56 vs. 2.01). After adjusting for health indicators (Model 2), most of these differences attenuated and were not statistically significant. However, Moluccans reported significantly fewer physiotherapist visits (rate ratio (RR) = 0.54) than the Dutch population. Further adjustments for the educational level and religious affiliation (Model 3) did not change these results for most healthcare services, except for hospital admissions (RR = 0.74).

## 4. Discussion 

This study explored differences in healthcare utilisation between Moluccans and the Dutch population. After adjusting for differences in health indicators, we found that Moluccans used most types of healthcare less frequently than the Dutch population. Of those who visited healthcare services, differences in the frequency of visits were generally small, except for a lower frequency of physiotherapist visits and hospital admissions among Moluccans. 

This study has four limitations that need consideration when interpreting the results. First, the response rate to the survey was only 24%. This is lower than the response rate of about 40% in similar studies among large minority groups in the Netherlands [17]. The response rate in our study was low due to community-level factors such as distrust within the local communities [8]. This observed distrust reinforced pre-existing reluctance among Moluccans to reveal personal information to other people. For the current analysis, an important question is whether the low participation rate of Moluccans may have caused an underestimation of the rate of healthcare utilisation. In theory, this could happen if Moluccans with health problems were less likely to participate. However, this is unlikely considering the relatively high prevalence of health problems observed in this survey among Moluccans. 

Second, our results could be influenced by a response bias. During the fieldwork in Moluccan districts, we observed a strong sense of respect for privacy. Privacy-sensitive information such as health-related issues may perhaps not be easily disclosed to those outside the community. This might make Moluccan respondents reluctant to report their use of healthcare. However, the high rates of reporting of different types of health problems suggest that those Moluccans who participated in this survey did not feel restrained in revealing health-related information.

Thirdly, the discrimination of Moluccans might have an impact on their use of healthcare services. Several studies showed that Moluccans, in the first decades after migration, felt discriminated against in daily life, including in the labour market and within workplaces, and prevented from full participation in the Dutch society [18,19,20]. Even today, some of the third-generation Moluccans report experiencing discrimination in everyday life [21]. This could reflect on their lower use of Dutch healthcare services. 

Fourthly, it would be of more interest, in this study, to stratify healthcare use by generation. However, the number of respondents were too small for such an analysis. In a previous study about mortality among Moluccans compared to the Dutch, it was possible to stratify by generation [12]. The largest mortality differences were found among the first-generation compared to the second- and third-generation Moluccans. We might expect a similar difference between Moluccan generations in healthcare use.

Our results could be compared to estimates based on registered healthcare claims. For example, a Dutch study using data from one insurance company found that, over a one-year period (2010), 79.9% of Moluccans aged 55 years and older used GP services as compared to 82.7% of the Dutch population [22]. De Back et al. reported fewer GP visits among Moluccan patients with a cardiovascular disease compared to Dutch patients, whereas they were equally likely to visit an outpatient medical specialist, such as the cardiologist and neurologist [9]. An additional analysis of the latter database showed that 88.2% of the Moluccans consulted a GP compared to 89.1% of the Dutch population over a two-year period (2009–2010). Thus, although insurance data also suggested a lower use of healthcare services by the Moluccan population, the magnitude of differences seemed smaller than observed in our health survey. 

Moluccans show substantially lower healthcare utilisation after accounting for need. This might seem surprising considering that Moluccans moved to the Netherlands over 60 years ago; it might be expected that, especially, those born in the Netherlands would now be familiar with the Dutch healthcare system and the society in general. Moreover, basic healthcare insurance is compulsory in the Netherlands, implying that there is no substantial financial barrier to use healthcare services. Possibly, the lower healthcare utilisation of Moluccans is related to demand-side, cultural factors related to the country of origin, such as health beliefs, family networks, and the use of traditional medicines. Each of these factors is discussed below.

Specific beliefs regarding the need for medical care among Moluccans may result in lower healthcare utilisation [23]. Due to the military profession of the first generation of Moluccan men, most Moluccan children had a strict upbringing, emphasising aspects such as fearlessness, toughness, and the ability to ignore pain [19,24,25]. A lack of interpretation by Moluccans of the serious nature of their symptoms may also play a role in this matter. As a result, many Moluccans may have learned to respond to physical discomfort by enduring suffering. For them, this may lead to postponing formal medical care when the first symptoms of a disease appear.

Strong family networks within Moluccan communities may influence the lower use of paid homecare services, especially housekeeping services, among Moluccans compared to the Dutch. Informal care provided by relatives is strongly embedded in the Moluccan culture [26,27,28,29]. Children are expected to provide care to their parents whenever it is needed. Although social norms are shifting among the younger generation of Moluccans [30], Moluccan elderly may still insist on receiving informal care instead of seeking nursing home care [30].

The preference for traditional medicine among Moluccans may have contributed to their lower use of prescribed and nonprescribed medications. In the present study, 48% of the Moluccan respondents reported to use *minyaks*, i.e., traditional oils used for several physical complaints such as stomach-aches, muscle aches, and for a cold. Additionally, 9% of the respondents used imported traditional herbs for similar purposes. A preference for traditional medicine, which is not equivalent to the use of “alternative” methods as preferred by the Dutch, has also been observed among other migrants from low/mid-income countries [31,32,33]. 

## 5. Conclusions

This study showed that, even after more than six decades of residence in the host country, the equal utilisation of healthcare services has not been achieved for Moluccans living in the Netherlands. Demand-side, culturally related factors, such as strong family networks, stoic health beliefs, and the use of traditional medicine, may contribute to the lower rates of healthcare utilisation. A particular challenge to healthcare for migrant groups is therefore to address such demand-based factors, both among those who arrived long ago and those who arrived more recently. Future qualitative research is needed to better understand which persisting mechanisms underlie these demand-based factors. Such research could focus in more depth on the existing health beliefs, strong family networks, and experienced discrimination among migrants.

## Figures and Tables

**Table 1:** Overview of the demographic characteristics of the Moluccan and Dutch populations (% and *N*).

Demographic Characteristics	Moluccan *N* = 715	Dutch *N* = 3417	Difference (*p*-Value)
Gender			0.743
men	48.1 (344)	47.4 (1621)	
women	51.9 (371)	52.6 (1796)	
Age group (in years)			<0.000
30–39	7.6 (54)	19.5 (668)	
40–49	15.4 (110)	27.6 (944)	
50–59	49.7 (355)	32.2 (1099)	
60–65	27.4 (196)	20.7 (706)	
Level of education			<0.000
Primary education	3.4 (24)	9.0 (307)	
Secondary education	44.1 (315)	23.9 (815)	
Secondary vocational education	32.9 (235)	30.1 (1027)	
Higher education	16.6 (119)	33.6 (1149)	
Missing cases (*N*)	22	119	
Religious affiliation			<0.000
Christian	91.6 (655)	46.2 (1579)	
Islamic	3.9 (28)	0.0 (1)	
Other	0.4 (3)	3.6 (122)	
No religious affiliation	3.8 (27)	46.2 (1579)	
Missing cases (*N*)	2	136	

The chi-Square test was used to test for differences between both populations. *p*-value ≤ 0.05 was significant.

**Table 2:** Age-standardised prevalence rates of health indicators of the Moluccan and Dutch populations (% and *N*).

Health Indicators	Total		
	Moluccan *N* = 715	Dutch *N* = 3417	Difference (*p*-Value)
Chronic disease			0.000
None	26.3 (188)	41.5 (1538)	
1	28.7 (205)	28.5 (978)	
2	17.2 (123)	14.9 (461)	
≥3	22.7 (199)	14.8 (440)	
Missing cases (*N*)	0	0	
Mental health			0.000
Good (score > 80)	49.1 (340)	60.3 (2002)	
Moderate (score 60–80)	39.6 (273)	28.4 (972)	
Poor (score < 60)	11.3 (78)	11.3 (364)	
Missing cases (*N*)	24	79	
Self-reported health			0.000
Excellent-very good	22.3 (158)	44.6 (1612)	
Good	58.8 (416)	43.9 (1445)	
Moderate-bad	18.9 (134)	11.5 (343)	
Missing cases (*N*)	7	17	
Disability			0.000
1 or more disabilities	20.9 (171)	13.9 (479)	
Missing cases (*N*)	4	2606	
Hearing	3.0 (15)	2.7 (81)	0.680
Missing cases (*N*)	10	22	
Eyesight	13.3 (94)	7.3 (221)	0.000
Missing cases (*N*)	6	18	
Mobility	10.0 (71)	6.0 (175)	0.000
Missing cases (*N*)	5	18	
Communication	0.1 (1)	0.3 (8)	0.624
Missing cases (*N*)	5	18	

The chi-square test was used to test for differences between both populations. *p*-value ≤ 0.05 was significant. Percentages were calculated without taking into account the missing cases.

**Table 3:** Age-standardised prevalence rates (%) and binary logistic regression of healthcare service utilisation of the Moluccan and Dutch populations (reference).

Healthcare Service	Total			Model 1		Model 2		Model 3	
	Moluccan *N* = 715 (%)	Dutch *N* = 3417 (%)	Difference (*p*-Value)	OR	95% CI	OR	95% CI	OR	95% CI
GP *	72.6	75.5	0.477	0.90	0.74–1.09	**0.73**	**0.59–0.90**	**0.67**	**0.53–0.84**
Outpatient medical specialist *	35.6	45.3	0.000	**0.68**	**0.57–0.82**	**0.52**	**0.42–0.63**	**0.50**	**0.41–0.62**
Dentist *	75.2	81.9	0.000	**0.66**	**0.54–0.82**	**0.72**	**0.58–0.90**	**0.65**	**0.51–0.83**
Physiotherapy *	22.5	30.0	0.000	**0.72**	**0.59–0.89**	**0.62**	**0.50–0.77**	**0.56**	**0.44–0.71**
Alternative medicine practitioner *	5.2	7.9	0.003	**0.66**	**0.45–0.97**	**0.61**	**0.41–0.90**	**0.57**	**0.38–0.87**
Hospitalisation *	7.1	6.8	0.205	1.08	0.77–1.52	1.05	0.74–1.50	0.94	0.64–1.38
Paid home care services *									
housekeeping	1.5	2.3	0.209	0.53	0.24–1.18	**0.34**	**0.15–0.77**	**0.37**	**0.15–0.87**
personal care	0.3	0.7	0.314	0.57	0.13–2.51	0.38	0.08–1.70	0.48	0.09–2.43
Medication utilisation ^#^									
prescribed	44.8	50.0	0.776	**0.81**	**0.68–0.97**	**0.60**	**0.49–0.73**	**0.63**	**0.51–0.79**
nonprescribed	27.5	38.0	0.000	**0.60**	**0.49–0.73**	**0.56**	**0.46–0.69**	**0.61**	**0.49–0.75**

Odds ratios (OR) are presented with the corresponding 95% confidence interval (95% CI). OR with a *p*-value ≤ 0.05 are presented in bold. The chi-square test was used to test for differences between both populations. A *p*-value ≤ 0.05 is significant. GP = general practitioner; * = healthcare service utilisation the past 12 months, and ^#^ = healthcare service utilisation the past 14 days. Model 1: adjusted for age and gender, Model 2: adjusted for model 1 and indicators of health, and Model 3: adjusted for model 2 and level of education and religious affiliation.

**Table 4:** Frequency (at least 1 visit) of healthcare service utilisation of the Moluccan and Dutch populations (reference). Persons with 0 visits were excluded from the analysis.

Healthcare Service	Study Populations			Model 1	Model 2	Model 3
	Moluccan (*N*)	Dutch (*N*)	Difference (*p*-Value)	RR 95% CI	RR 95% CI	RR 95% CI
General practitioner						
N of cases (at least 1 visit)	261	756				
Visits past 4 weeks ^#^	1.56 (sd. 1.03)	1.42 (sd. 1.04)	0.000	1.09 (0.97–1.23)	1.04 (0.92–1.18)	1.04 (0.90–1.21)
Outpatient medical specialist						
N of cases (at least 1 visit)	116	439				
Visits past 4 weeks ^#^	1.78 (sd. 1.34)	1.55 (sd. 1.28)	0.009	1.16 (0.98–1.37)	1.14 (0.96–1.36)	1.19 (0.99–1.44)
Dentist						
N of cases (at least 1 visit)	167	581				
Visits past 4 weeks ^#^	1.38 (sd. 1.68)	1.20 (sd. 0.57)	0.000	1.13 (0.96–1.32)	1.15 (0.97–1.36)	1.13 (0.94–1.36)
Physiotherapist						
N of cases (at least 1 visit)	153	71				
Visits past 12 months ^#^	12.67 (sd. 13.20)	26.06 (sd. 15.62)	0.000	**0.50 (0.47–0.54)**	**0.54 (0.50** **–** **0.58)**	**0.51 (0.46–0.55)**
Hospitalisation						
N of cases (at least 1 visit)	43	199				
Admissions past 12 months ^#^	1.56 (sd. 1.03)	2.01 (sd. 5.25)	0.425	0.80 (0.61–1.04)	0.82 (0.61–1.09)	**0.74 (0.55–1.00)**

The rate ratios (RR; poison regression analysis) are presented with the corresponding 95% confidence interval (95% CI). RR with a *p*-value ≤ 0.05 are presented in bold. ^#^ = Mean of all with at least 1 visit. Differences between both populations is calculated with the Mann-Whitney test. *p*-value ≤ 0.05 is significant. Model 1: adjusted for age and gender, Model 2: adjusted for model 1 and indicators of health, and Model 3: adjusted for model 2 and level of education and religious affiliation.
